# Systems Study on the Antirheumatic Mechanism of Tibetan Medicated-Bath Therapy Using Wuwei-Ganlu-Yaoyu-Keli

**DOI:** 10.1155/2017/2320932

**Published:** 2017-09-26

**Authors:** Tianhong Wang, Jian Yang, Xing Chen, Kehui Zhao, Jing Wang, Yi Zhang, Jing Zhao, Yang Ga

**Affiliations:** ^1^School of National Medicine, Chengdu University of TCM, Chengdu, China; ^2^School of Pharmacy, Second Military Medical University, Shanghai, China; ^3^Department of Mathematics, Logistical Engineering University, Chongqing, China; ^4^Institute of Interdisciplinary Complex Research, Shanghai University of Traditional Chinese Medicine, Shanghai, China; ^5^Tibetan Traditional Medical College, Lhasa, China

## Abstract

In clinical practice at Tibetan area of China, Traditional Tibetan Medicine formula Wuwei-Ganlu-Yaoyu-Keli (WGYK) is commonly added in warm water of bath therapy to treat rheumatoid arthritis (RA). However, its mechanism of action is not well interpreted yet. In this paper, we first verify WGYK's anti-RA effect by an animal experiment. Then, based on gene expression data from microarray experiments, we apply approaches of network pharmacology to further reveal the mechanism of action for WGYK to treat RA by analyzing protein-protein interactions and pathways. This study may facilitate our understanding of anti-RA effect of WGYK from perspective of network pharmacology.

## 1. Introduction

Rheumatoid arthritis (RA) is a chronic inflammatory autoimmune disease, which primarily attacks the synovial joints and leads to the destruction of the cartilage and bone. It can also affect multiple organs throughout the body [1]. Currently RA cannot be completely cured. The aim of treatment is to eliminate the inflammation, assuage the pain, control disease activity, prevent joint damage, and retard disease progression, so as to enhance patient's quality of life.

Tibetan Plateau is an area where RA commonly occurs. Tibetan medicated-bath therapy is one of the efficient treatments to RA in the Tibetan Medicine [2], in which the Tibetan formula Wuwei-Ganlu-Yaoyu-Keli for the bath therapy has been included in the year 1995 version of the Tibetan Medicine Standard [3] issued by the Chinese Ministry of Health. However, there is not enough research into the mode of action of Tibetan medicated-bath therapy; thus its mechanism in the modern biomedical background is not well understood.

In this work, we conducted a systems study to explore the anti-RA mechanism of Tibetan medicated-bath therapy using Wuwei-Ganlu-Yaoyu-Keli (WGYK) as compared with oral medicine dexamethasone acetate (DMA) and topical creams Qing Peng ointment (QPO) used clinically in the treatment of RA [4, 5]. Adjuvant arthritis (AA) in rat has been widely used as an experimental model that shares some features with human RA, such as swelling, cartilage degradation, and loss of joint function [6]. Treating AA model rats by WGYK with the warm water bath, DMA through the mouth, and QPO external painting, respectively, we compared the effects of these different treatments to relieve foot swelling and applied gene chip technology to detect differentially expressed genes in synovial cells under each treatment. Then we used approaches of network pharmacology to identify signaling pathways and subnetworks influenced by the AA modeling disease and regulated by different drugs [7]. At last, we checked the overlaps between the pathways or subnetworks to deduce the effects of the drugs on the disease.

## 2. Materials and Methods

### 2.1. Reagents and Drugs

The Freund's complete adjuvant was obtained from Sigma Company. WGYK was purchased from Qizheng Tibetan Medicine Limited Company. DMA was produced by Zhejiang Xianju Pharmaceutical Limited Company. QPO was purchased from Jinhe Tibetan Medicine Limited Company.

### 2.2. Animal Experiment

We used 40 ± 5 days healthy male SD rats (Chengdu Dashuo Biological Technology Co. Ltd.) with a body weight of 200 ± 20 g. The experiment as follows was carried out after one week of adaptive feeding in the animal laboratory of Chengdu University of Traditional Chinese Medicine.

1. The rats were randomly divided into seven groups, each of which included ten animals, that is, control group (C), adjuvant arthritis group (AA), dexamethasone acetate group (DMA), Qing Peng ointment group (QPO), low dose Wuwei-Ganlu-Yaoyu-Keli group (WGYKl), moderate dose Wuwei-Ganlu-Yaoyu-Keli group (WGYKm), and high dose Wuwei-Ganlu-Yaoyu-Keli group (WGYKh).
2. The rats in the six groups AA, DMA, QPO, WGYKl, WGYKm, and WGYKh were injected with 0.1 mL of Freund's complete adjuvant in the skin of right rear toe, to establish the model of adjuvant arthritis rats. Control animals were similarly injected with normal saline.
3. Two weeks after the injection, the rats in seven groups were treated in different way by drugs or dipping bath. Specifically, the rats in the groups of C, AA, WGYKl, WGYKm, and WGYKh were treated by bath therapy of different liquids. We fixed the rats at wood cylindrical bathtubs of diameter 30 cm and height 20 cm and soaked their legs into 2 L liquid at 40 ± 2°C. The bath therapy was conducted 30 min one day. A course of treatment lasted 7 days. We conducted 4 courses. After each course, the treatment was stopped for 2 days. The liquid used in the C and AA groups was fresh warm water, while the WGYKl, WGYKm, and WGYKh groups used medical solution of WGYK whose concentrations were 2.95 g/L, 5.90 g/L, and 11.80 g/L, respectively. In the DMA group, rat stomachs were perfused with dexamethasone at the dose of 0.15 mg/kg. Rats in the QPO group were painted with appropriate amount of Qing Peng ointment. The treatment frequency and course of treatment for groups DMA and QPO were the same as those of medicated-bath treatment groups.

### 2.3. Measurement of Swelling Degree of Foot

The volume of each rat's right rear foot was measured twice before making animal model, while the average of the two measures was taken as the basic value. Using the same method, we measured the swelling extent of the feet in each group of rats at the fifth day, the ninth day, the thirteenth day, and the day before the treatment. After two weeks that the rats were administered drugs, we measured the foot volumes of the inflammatory side (right) at the first, second, third, and fourth week, respectively, and calculated the primary foot swelling as follows:

ΔmL = the average foot volume after inflammation − the average foot volume before inflammation.

The data was processed with SPSS 19 statistical software. All values were expressed as means ± standard errors. Single factor analysis of variance was used to compare the difference between groups. The variance was not homogeneous, and the rank sum test was used. A *P* value < 0.05 was considered statistically significant.

### 2.4. Microarray Experiment and Significantly Expressed Genes

#### 2.4.1. Sampling of the Synovial Tissue

The rats were sacrificed under anesthesia, along the median incision of the skin of right rear ankle joint (inflammatory side). Then we exposed about 3 × 3 cm^2^ region in the center of the ankle joint and stripped part of smooth and bright yellow synovial tissue. At last we randomly selected three cases of synovial tissue samples from each of the seven groups.

#### 2.4.2. Microarray Experiment

The synovial tissues were washed by saline and ground in liquid nitrogen. Then the RNA was extracted using the Trizol reagent (Life technologies, Carlsbad, CA, US). Further, quality control and purity of isolated total RNA were performed by UV spectrophotometer (Beijing Kai'Ao company), agarose gel electrophoresis, and Agilent Bioanalyzer 2200 (Agilent Company, USA) and the qualified RNA samples were subpacked and stored at −80°C for further use. RNA profiling was performed by Guangzhou RIBOBIO Company in China. Genes whose |FoldChange| > 0.585 (i.e., log⁡2 value of 1.5) and *P*≤ 0.05 were considered as differentially expressed [8, 9]. Here the FoldChange is the log⁡2 ratio of average expression intensities between the treatment and control group.

### 2.5. Data of Human Gene Association Network

Gene association network links genes or encoded proteins by their functional interplays, including direct physical binding and indirect interaction such as being involved in the same cellular process. Here we utilized the human functional linkage network (FLN) constructed by Linghu et al. [10]. FLN is a densely connected weighted network composed of 21,657 genes and 22,388,609 edges, in which nodes represent genes, and there is an edge if two genes participate in a common biological process. The edge weight is a probabilistic confidence score of the linkage. We normalized the original edge weight to the interval [0,1].

### 2.6. Data of FDA Approved Anti-Ra Drugs and Their Target Proteins

Four classes of drugs are used clinically for the treatment of RA. They are nonsteroidal anti-inflammatory drugs (NSAID) such as flurbiprofen, disease-modifying antirheumatic drugs (DMARDs) such as sulfasalazine, glucocorticoids such as cortisone acetate, and biological response modifiers such as etanercept and abatacept. The data of FDA approved anti-RA drugs and their targets were downloaded from the DrugBank database version 5.01 [11], which was updated in July of 2016. We searched the DrugBank database with a keyword “rheumatoid arthritis” and extracted all of the FDA approved anti-RA drugs and their corresponding targets. In this way, 51 FDA approved anti-RA drugs and corresponding 82 protein targets were collected.

### 2.7. Pathway Enrichment Analysis

We used pathway enrichment analysis [12] to identify pathways significantly influenced by a group of differentially expressed genes. Hypergeometric cumulative distribution was applied to quantitatively measure whether a pathway was more enriched with the group of genes than would be expected by chance [13]. In our case, if all pathways under study include *N* distinct genes, in which *K* genes are differentially expressed genes, for a randomly chosen pathway which owns *n* genes, the probability that we can find *i* differentially expressed genes in this pathway by chance obeys hypergeometric distribution:

(1) $$\begin{array}{c} f\left(\;i\;\right)=\frac{\left(\begin{array}{c} K\\ i \end{array}\right)\left(\begin{array}{c} N-K\\ n-i \end{array}\right)}{\left(\begin{array}{c} N\\ n \end{array}\right)}. \end{array}$$

Then the probability of getting at least *k* differentially expressed genes in this pathway by chance can be represented by hypergeometric cumulative distribution defined as *P* value:

(2) $$\begin{array}{c} P=1-\overset{k-1}{\underset{i=0}{\sum }}f\left(\;i\;\right)=1-\overset{k-1}{\underset{i=0}{\sum }}\frac{\left(\begin{array}{c} K\\ i \end{array}\right)\left(\begin{array}{c} N-K\\ n-i \end{array}\right)}{\left(\begin{array}{c} N\\ n \end{array}\right)} \end{array}$$

Given significance level *α*, a *P* value smaller than *α* implies a low probability that the *k* differentially expressed genes appear in the pathway by chance; that is, this pathway can be regarded as significantly influenced by these genes.

### 2.8. Scoring Network Effect of a Group of Differentially Expressed Genes

A group of differentially expressed genes under a specific condition, such as a disease status or a drug treatment, could exert their impact on other genes through network links. For each gene *i* in the human gene association network FLN, we quantified the influence of differentially expressed genes by a network effect score. In general, the higher score a gene receives, the deeper and more pronounced it is affected by the disease or drug. Specifically, a node's score is defined as follows:

(3) $$\begin{array}{c} S_{i}=\overset{n}{\underset{j=1}{\sum }}w_{j}^{\left(v\right)}W_{ij}^{\left(e\right)}, \end{array}$$

where *n* is the number of nodes in the network and *w*_*j*_^(*v*)^ is the weight of the node *j* defined as absolute value of log⁡2 ratio of the expression level if the corresponding gene is differentially expressed; otherwise it is zero. *W*_*ij*_^(*e*)^ is the linkage weight connecting the genes *i* and *j*, and it is defined as 1 when *i* = *j*.

### 2.9. Construction of Condition Specific Network

For a specific condition, such as a disease status or a drug treatment, we defined a condition specific network as a subnetwork of human gene association network consisting of nodes with high network effect scores. We sorted the effect scores under this condition decreasingly and collected certain fraction of top genes in the rank list. Then these genes and their links were extracted from human gene association network to construct the condition specific network. In this way, we constructed a network impacted by a disease or regulated by a drug, respectively.

### 2.10. Generating Random Counterparts of Differentially Expressed Genes Under the Treatment of a Drug

For the group of differentially expressed genes under the treatment of a drug, we randomly selected the same number of genes in the background network as a random counterpart. We assigned the values of expression level of the differentially expressed genes to the genes in the counterpart randomly. Repeating this process a sufficiently large number of times gave us a set of random counterparts of the differentially expressed genes, which we used as a random reference of gene expression levels for this drug's effect.

## 3. Results and Discussion

### 3.1. Evaluation of Drug Effects by Foot Swelling Degrees

**Figure 1:**
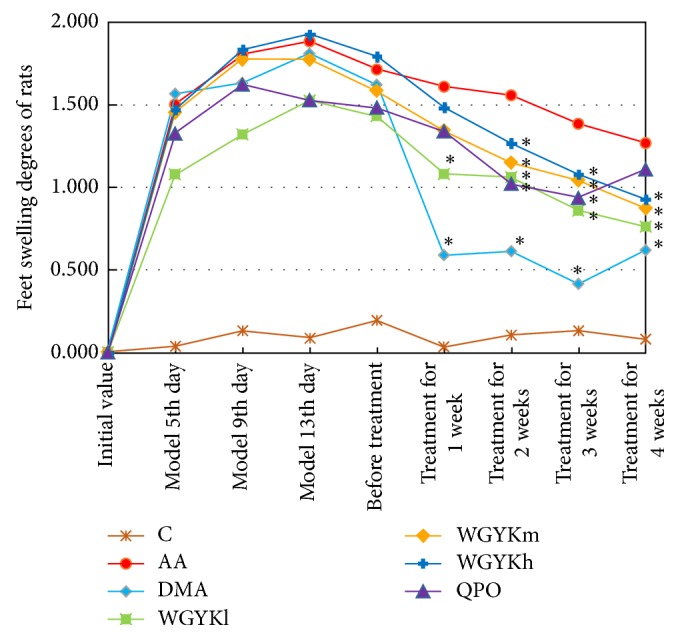
Changes in foot swelling degree of rats in each group: normal control (C), adjuvant arthritis (AA), dexamethasone (DMA), high dose Wuwei-Ganlu-Yaoyu-Keli (WGYKh), moderate dose Wuwei-Ganlu-Yaoyu-Keli (WGYKm), low dose Wuwei-Ganlu-Yaoyu-Keli (WGYKl), and Qing Peng ointment (QPO). Each point represents the mean ± SE. The points with black *∗* sign have significant difference (*P* value < 0.05) from the adjuvant arthritis group at the respective time.

Foot swelling degrees are used as apparent indicators of arthritis to evaluate the arthritic progression of adjuvant-induced arthritis [14]. It is known that redness and swelling of the joints usually appear at the onset of arthritis. We found that the foot volumes of the control rats were at a stable level during the experimental period; meanwhile the foot volumes of the rats in the AA, DMA, WGYKh, WGYKm, WGYKl, and QPO groups reached peak values about 2 weeks after adjuvant injection. After one week's treatment, the foot swelling degree of DMA group decreased sharply, and the WGYKh, WGYKm, WGYKl, and QPO groups decreased significantly compared with the AA group. In the treatment of the second to third week, the foot swelling of all groups showed a downward trend, in which the foot swelling degree of rats in the DMA group tended to be stable, and the foot swelling degree of rats in the WGYKm, WGYKh, WGYKl, and QPO groups continued to decline. In the fourth week, DMA and QPO group slightly rebounded (Figure 1).

This experiment suggests that all the drugs under study have significant effect of alleviating swelling in AA rats, in which DMA shows best effect but rebound appears if the treatment lasts four weeks.

### 3.2. Differentially Expressed Genes in the AA Model and under the Treatment of Drugs

The notable feature of RA is progressive joint damage caused by chronic synovitis. A large number of activated cytokines are found in the joints of RA patients, which are the key mediator of inflammation and play an important role in the generation of joint injury and other complications. Many researches in the past decades have revealed that cytokines such as tumor necrosis alpha (TNF-a) and interleukins 1, 6, and 15 (IL1, IL6, and IL15), as well as immune-mediated inflammatory signaling pathways such as JAK-STAT, NF-*κ*B, and MAPK signaling pathway, are deeply involved in the occurrence and development of RA [15]. Thus we took all genes on three typical inflammatory pathways, JAK-STAT, NF-*κ*B, and MAPK signaling pathway, as background genes to perform microarray experiment. There are totally 470 distinct genes on these three pathways. We checked the expression levels of these 470 genes in synovial tissues of 7 groups of experimental rats, that is, control group, AA model group, DMA treatment group, QPO treatment group, low dose WGYK treatment group, moderate dose WGYK treatment group, and high dose WGYK treatment group. The expression levels of all genes in AA model group were compared against the expression levels in the control group, while the expression levels of all genes in each drug treatment group were compared against the expression levels in the AA model group. In this way, we totally had six different conditions under study, that is, one condition of disease denoted as AA and five conditions of drug treatment denoted as DMA, QPO, WGYKl, WGYKm, and WGYKh, respectively. Under each condition, genes whose mean expression ratios between treatment group and control group were greater than 1.5 or less than 0.667 (1/1.5), as well as *P*≤ 0.05, were considered as differentially expressed.

**Figure 2:**
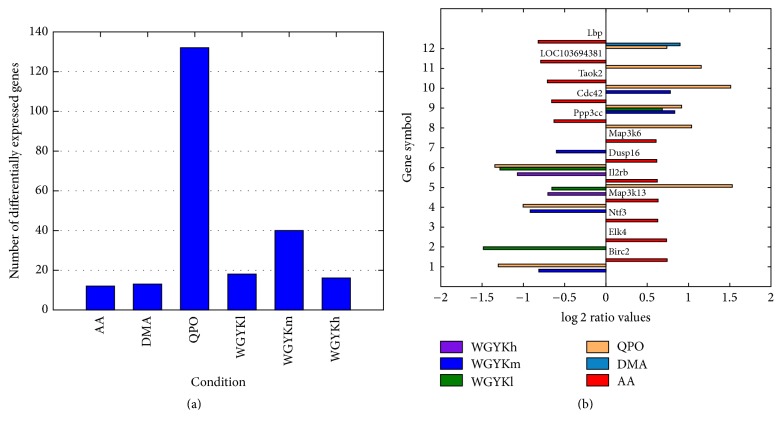
(a) Number of differentially expressed genes under different conditions. (b) Overlaps of differentially expressed genes in AA model and those under the treatment of different drugs with their corresponding log⁡2 ratio values.

In Figure 2(a) we show a comparison of the number of differentially expressed genes in the AA model and under the treatment of the five drug types. It can be seen that QPO influences the most genes, then followed by moderate dose of WGYK.

To see if the abnormally expressed genes in the disease status could be rectified by the drugs, in Figure 2(b) we show log⁡2 ratio values of all the 12 differentially expressed genes in the AA model and their differential expression ratios under the treatment of our drugs. It shows that, in almost all cases, the drugs could directly upregulate some genes lowly expressed in the disease status and downregulate some highly expressed genes in the disease status, with the only exception of Il2rb by QPO. This suggests the therapeutic effects of the drugs to some degree. Similar as in Figure 2(a), QPO regulates the most number of abnormal genes in disease and the moderate dose WGYK ranks the second. The moderate dose WGYK rectifies about half of the 12 abnormally expressed genes in the AA modeled disease. However, we also notice that although DMA shows the best anti-RA performance in our animal experiment, it only directly influences one abnormally expressed gene in the AA model, indicating that the drugs may exert their functions on disease by regulating other genes.

### 3.3. Significantly Regulated Pathways in the AA Model and under the Treatment of Drugs

To deduce the possible pathways affected by AA and the drugs, we, respectively, mapped the differentially expressed genes under different conditions onto KEGG pathways of basic biological process [16], including pathways in metabolism, organismal systems, cellular processes, environmental information processing, and genetic information processing. We conducted pathway enrichment analysis to identify the pathways significantly affected by the disease and the drugs through calculating *P* values for each of the pathways. Taking pathways with values of *P* < 0.05 as significantly impacted pathways, we identified pathways affected by the corresponding disease or drugs.

It comes out that only 4 pathways, that is, apoptosis, MAPK, VEGF, and T-cell receptor signaling pathway, are significantly enriched with differentially expressed genes under the condition AA, suggesting that they are affected by the AA modeling RA disease and could be dysfunctional in the disease status. Meanwhile much more pathways are significantly enriched with differentially expressed genes under most of the drug treatment conditions. In detail, the numbers of pathways affected by the treatment of QPO, WGYKm, WGYKl, WGYKh, and DMA are 25, 14, 10, 7, and 4, respectively.

The four pathways dysfunctional in the AA model have been reported to be related to the pathology of RA. Actually, earlier experiments have revealed that apoptotic pathways are defective in RA synovial tissue, resulting in “apoptosis-resistance” phenomena that few apoptotic cells can be detected in joints of RA patients [17]. MAPK signaling plays a significant role in the regulation of immune-mediated inflammatory responses and therefore it gets involved in the process of several autoimmune diseases including RA [18]. Vascular endothelial growth factor (VEGF) has been known to play angiogenic, inflammatory, and bone destructive roles in RA. In the adaptive immune response process, T-cell receptors participate in the activation of T-cells in response to foreign pathogens specifically and sensitively. Altered T-cell receptor signaling could contribute to human autoimmune arthritis, including RA [19]. Our pathway enrichment analysis shows that the five types of drugs regulate two to four of these four pathways influenced by the AA modeling disease. Specifically, QPO acts on all of these pathways, while moderate dose WGYK regulates three of them, suggesting anti-RA effect of these drugs.

Although only four pathways were detected to be enriched with differentially expressed genes in the AA model, much more pathways were identified being affected by the treatment of our drugs, many of which have been known being deeply involved in the initiation and progress of RA. For instance, Jak-STAT and NF-kappa B signaling pathways are proinflammatory cytokine mediated pathways related to immune-mediated inflammation and following damage of cartilage and bone in RA [18]. The osteoclast differentiation pathway maintains bone density and structure through a balance of bone resorption by osteoclasts and bone deposition by osteoblasts. Its dysfunction may disturb this balance. Both QPO and WGYKm regulate these three pathways, indicating that they are conducive to disease remission of RA.

**Figure 3:**
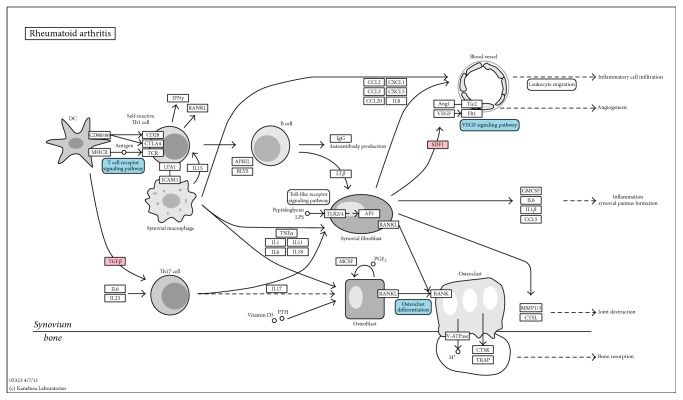
Regulations of WGYKm on RA pathway. Pink boxes represent differentially expressed genes under the treatment of WGYKm that appear on the RA pathway, while blue boxes represent WGYKm regulated pathways involved in the RA biological process. The original pathway map was downloaded from the KEGG database.

At last, to see how moderate dose WGYK acts on the biological processes of RA, we mapped differentially expressed genes under the treatment of WGYKm, as well as pathways enriched with these genes onto the RA pathway in the KEGG database (Figure 3) [16]. It is found that WGYKm intervenes in three important pathways along the RA developing process, that is, T-cell receptor signaling pathway, VEGF signaling pathway, and osteoclast differentiation pathway. In addition, WGYKm influences two genes on the RA pathway. These results also suggest the therapeutic effect of WGYKm on RA.

### 3.4. Drug's Effects on AA Influenced Gene Association Network

To explore the relationships between the differentially expressed genes in different conditions and drug targets of FDA approved anti-RA drugs, we studied these genes in the context of human gene association network. We mapped all the differentially expressed rat genes onto their orthologs in human beings using Inparanoid database [20]. Thus we could check if these differentially expressed genes encode target proteins. It turns out that although 82 distinct proteins are known to be targeted by FDA approved anti-RA drugs, only three and one of their genes are differentially expressed under the treatment of QPO and moderate dose WGYK, respectively. No target genes are differentially expressed in the AA model and under the treatment of DMA and WGYK at low and high doses.

**Figure 4:**
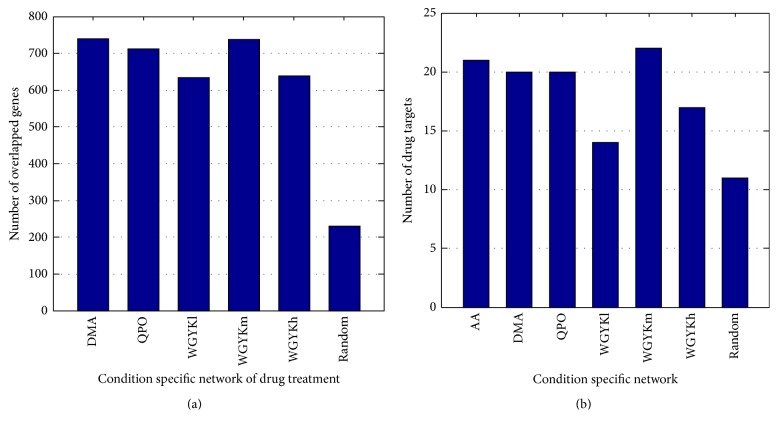
(a) Number of overlapped genes in different drug regulated network with the disease influenced network. (b) Number of drug targets for FDA approved anti-RA drugs included in disease influenced network and different drug regulated networks.

For each condition, that is, the AA model or drug treatment, we applied (3) to score the impact of its differentially expressed genes on each of the 21657 genes in the human gene association network. The higher the score is, the greater the gene is influenced by the group of differentially expressed genes. Thus a subnetwork consisting of high-score genes could be considered as a condition specific network influenced by this condition. We selected genes whose scores were top highest 1000 of the 21657 genes, that is, about top 5% of all genes in the whole network, to construct its condition specific network. In this way, we obtained one disease influenced network and five drug regulated networks, each of which owns 1000 nodes.  Figure 4(a) shows how each drug regulated network overlaps with the disease influenced network. It can be seen that DMA, QPO, and moderate dose WGYK regulate significantly much more genes located at the disease influenced network than WGYK at low and high dose do, suggesting that they may have better therapeutic performance on AA.

Then, we checked how many target genes of FDA approved anti-RA drugs were included in each condition specific network. From Figure 4(b) we can see that about one-fourth of these target genes are included in the network at condition AA, DMA, QPO, and WGYKm, significantly more enriched than in the network at condition WGYKl and WGYKh. In addition, by checking the overlapped target genes in different condition specific networks, we found that DMA, QPO, and moderate dose WGYK regulated 16 common target genes appearing in the disease influenced network, including the most important target gene PTGS2 for nonsteroidal anti-inflammatory agents and TNF for biotech agents. These results further suggest the better performance of DMA, QPO, and moderate dose WGYK to act against AA.

We generated 100 groups of random counterpart for the 39 differentially expressed genes under the treatment of moderate dose WGYK. For each counterpart set, we repeated the two processes illustrated above. Thus we got the average number of overlapped genes affected by the counterpart genes and the AA disease, as well as the average number of drug targets for FDA approved anti-RA drugs included in the network influenced by the counterpart genes. The averages for the 100 sets of counterpart are shown in the last bars in Figures 4(a) and 4(b), respectively. These values are significantly smaller than corresponding values of the drugs, further verifying the effect of the drugs to AA.

### 3.5. Gene Association Network Impacted by AA Disease and WGYKm

To see how moderate dose WGYK acts on the gene association network affected by AA disease, we constructed an overlapped network of the AA affected and WGYKm regulated networks. As illustrated in the last section, both of the networks include 1000 nodes which obtained the highest impact scores from the differentially expressed genes, in which 736 nodes are overlapped. We mapped these 736 genes into the background network FLN and extracted all links between them which have confidence score larger than 0.3. Among the 22,388,609 edges of the FLN network, only 9095 have confidence score larger than 0.3, taking a percentage of around 0.04%. Thus the constructed network is a high confidence gene association network regulated by AA disease and WGYKm. This network owns 292 nodes and 448 edges. It has 22 connected clusters, in which the largest one (called giant connected component) includes 231 nodes and only two other clusters have more than 4 nodes. We will focus on these 3 connected clusters.

**Figure 5:**
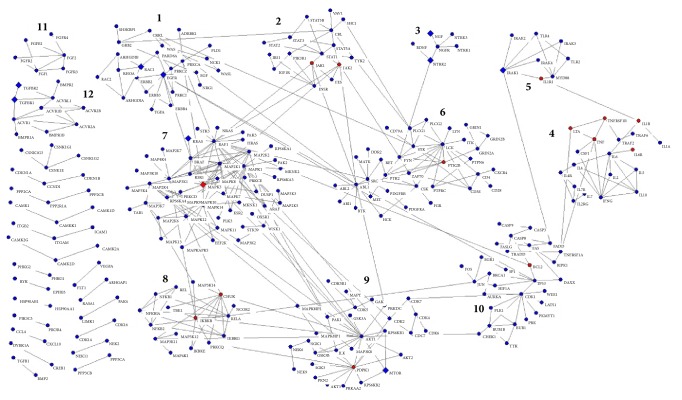
A gene association network regulated by AA disease and WGYKm, which includes high confidence links. The giant connected component of the network was decomposed into modules by Louvain algorithm. Diamond nodes are differentially expressed genes under the treatment of WGYKm, while red nodes are drug targets of FDA approved anti-RA drugs.

To investigate the biological functions of this network, we applied Louvain algorithm to decompose the giant connected component of the network into topological modules [21], so that there are much more links within modules than between modules. As shown in Figure 5, together with the other 2 larger connected clusters, this network can be considered as having 12 topological modules. Since it has been suggested that topological modules in molecular networks usually correspond to relatively independent biological functions [22], we conducted Gene Ontology (GO) enrichment analysis for each module [23]. The Gene Ontology Consortium is organized in a hierarchical way, from high level for generally descriptive terms to very low level for highly specific terms. For each module, we selected the GO terms with the highest statistical significance and the lowest GO levels, which can represent the major and specific functions of the module (see Table 1). Table 1 suggests that most modules that WGYKm acts on participate in the regulation of immune process, including pathogen recognition, proinflammatory response and inflammatory signaling in innate immune defenses (modules (1), (2), (4), (5), (6), (7), (8), and (12)), innate immune response (modules (5) and (8)), and adaptive immune response (module (4)). WGYKm also regulates cell proliferation and differentiation by influencing modules (3), (10), and (11).

Figure 5 shows that half of the modules include differentially expressed genes under the treatment of WGYKm, while 8 modules own genes that encode drug targets for FDA approved anti-RA drugs. We list these drug targets and corresponding anti-RA drugs in Table 2. Targets of 3 classes of anti-RA drugs, nonsteroidal anti-inflammatory drugs (NSAID), disease-modifying antirheumatic drugs (DMARDs), and biotechnology agents are included in different modules of the network. This result also suggests WGYKm's effect in anti-inflammation and regulation of immune process.

## 4. Conclusions

This work systematically studies the anti-RA mechanism of Tibetan medicated-bath therapy using Wuwei-Ganlu-Yaoyu-Keli. First, we performed animal experiment to verify that the bath therapy by different doses of WGYK exhibited similar effect of relieving foot swelling of adjuvant arthritis model rats as positive contracts dexamethasone and Qing Peng ointment did. Then, based on differentially expressed genes in the disease status and under the treatment of different drugs, we investigated the effects of the bath therapy on RA in the contexts of single genes, pathways, and networks, respectively. We found that the drugs could directly upregulate some lowly expressed genes and downregulate some highly expressed genes in the AA modeling disease status, in which moderate dose WGYK rectified about half of the 12 abnormally expressed genes in the disease status. Our pathway enrichment analysis revealed that moderate dose WGYK regulated three of the four pathways influenced by the disease. It also intervened in three of the five important pathways along the RA developing process recorded in the KEGG database. By scoring the impacts of abnormally expressed genes on all genes in the human gene association network, we constructed subnetworks influenced by the disease and regulated by the drugs, respectively. It comes out that the subnetwork regulated by moderate dose WGYK has more than 70% nodes overlapped with the subnetwork influenced by the AA modeling disease. In addition, 16 common target genes of FDA approved anti-RA drugs appear in the disease influenced subnetwork and the subnetworks regulated by moderate dose WGYK, dexamethasone, and Qing Peng ointment, including the most important target gene PTGS2 for nonsteroidal anti-inflammatory agents and TNF for biotech agents. Finally, we constructed a gene association network regulated by AA disease and WGYKm, which only includes high confidence links. Our GO analysis for topological modules of this network suggests that WGYKm performs its therapeutic effect on RA by regulating immune process, as well as cell proliferation and differentiation. All these results support the anti-RA effect of Tibetan medicated-bath therapy using Wuwei-Ganlu-Yaoyu-Keli at moderate dose.

This work applies network approach to explain WGYK's antirheumatic effect. It may shed light on the study about the pharmacology of Tibetan medicated-bath therapy and promote the development of traditional medicine.

## Figures and Tables

**Table 1:** Selection of the most significantly enriched and specific GO terms in the network modules.

Module	GO term (biological process)	Level	Total genes	Mapped genes
(1)	Positive regulation of interleukin-5 secretion	9	14	13
	Positive regulation of interleukin-13 secretion	9	14	13
	Positive regulation of T-helper 2 cell cytokine production	11	15	13
	Positive regulation of interleukin-10 secretion	9	15	13

(2)	JAK-STAT cascade	5	57	12
	JAK-STAT cascade involved in growth hormone signaling pathway	9	26	8
	Cytokine-mediated signaling pathway	6	406	15

(3)	Positive regulation of neuron differentiation	7	342	6
	Positive regulation of neurogenesis	7	472	6
	Positive regulation of neuron projection development	8	208	5

(4)	Positive regulation of leukocyte differentiation	6	137	19
	Positive regulation of hemopoiesis	6	166	19
	Regulation of adaptive immune response based on somatic recombination of immune receptors built from immunoglobulin superfamily domains	3	127	16

(5)	MyD88-dependent toll-like receptor signaling pathway	8	92	11
	Innate immune response-activating signal transduction	8	157	11

(6)	Immune response-activating cell surface receptor signaling pathway	7	350	33
	Antigen receptor-mediated signaling pathway	7	158	27

(7)	Positive regulation of MAPK cascade	10	512	39

(8)	Regulation of interleukin-12 biosynthetic process	6	16	9
	Positive regulation of type I interferon production	5	57	11
	Activation of innate immune response	7	170	13
	Positive regulation of cytokine biosynthetic process	10	87	11
	Regulation of I-kappaB kinase/NF-kappaB signaling	4	249	14

(9)	Cellular response to insulin stimulus	8	255	30
	Peripheral nervous system myelin maintenance	9	29	17

(10)	Apoptotic signaling pathway	5	310	18

(11)	Positive regulation of cell proliferation	3	964	16

(12)	Transmembrane receptor protein serine/threonine kinase signaling pathway	5	273	20
	Cellular response to growth factor stimulus	4	719	19

**Table 2:** Genes encoding drug targets for FDA approved anti-RA drugs that appear in the network modules.

Module	Drug target	Drug	Drug class
(2)	JAK1	Tofacitinib	DMARDs
	JAK2	Tofacitinib	DMARDs

(4)	IL1B	Canakinumab	Biotech agents
	IL6R	Tocilizumab	Biotech agents
	LTA	Etanercept	Biotech agents
	TNF	Etanercept	Biotech agents
		Adalimumab	Biotech agents
		Infliximab	Biotech agents
		Golimumab	Biotech agents
		Certolizumab pegol	Biotech agents
		Chloroquine	DMARDs
	TNFRSF1B	Etanercept	Biotech agents

(5)	IL1R1	Anakinra	Biotech agents

(6)	PTK2B	Leflunomide	DMARDs

(7)	MAPK3	Sulindac	NSAIAs

(8)	CHUK	Sulfasalazine	DMARDs
	IKBKB	Sulfasalazine	DMARDs
		Auranofin	DMARDs

(9)	PDPK1	Celecoxib	NSAIAs

(10)	BCL2	Ibuprofen	NSAIAs
